# Errors of multiple exponence in child English: a study of past tense formation

**DOI:** 10.1007/s11525-024-09434-x

**Published:** 2024-12-05

**Authors:** Johannes Hein, Imke Driemel, Fabienne Martin, Yining Nie, Artemis Alexiadou

**Affiliations:** 1 Department of German Studies and Linguistics, Humboldt-Universität zu Berlin, Unter den Linden 6, Berlin, 10099 Germany; 2 Department of Language and Linguistic Science, University of York, Heslington, York, YO105DD, UK; 3 Institute for language sciences, Utrecht University, Trans 10, Utrecht, 3512 JK The Netherlands; 4 Department of Linguistics and Language Development, San José State University, 1 Washington Sq, San Jose, CA 95192 USA; 5 Leibniz-Zentrum Allgemeine Sprachwissenschaft (ZAS), Pariser Str. 1, Berlin, 10719 Germany

**Keywords:** Overregularization, Multiple exponence, Overtensing, L1 acquisition, Distributed morphology, Allomorphy, Secondary features

## Abstract

It is well known that children produce non-adult-like forms during language acquisition. Among these are errors where in the fashion of multiple exponence the child overtly marks an underlying feature or category more than once. In addition, children also produce errors where features that are marked fusionally with one form in the target language are marked separately with more than one form by the child. This paper is concerned with such errors in the domain of English past tense. We present a comprehensive corpus study investigating the frequencies and distribution of different error types, combining both overregularization and overtensing errors, which have previously been studied separately. We then propose an analysis based on Generalized Head Movement (Arregi & Pietraszko, 2021) and Distributed Morphology arguing that errors can be derived from two occasionally occurring underlying mistakes: negligence of secondary features and omission of obliteration. We show how these two mistakes and their interaction can account for the overall differences in error rates and distributions between different error types as well as across different verbs.

## Introduction

During language acquisition, children produce errors of omission by which they do not externalize linguistic material required to be present in the adult language. They also produce another type of error by which they overtly pronounce material that is usually not realized in the standard adult language. This type is referred to as ‘undercompression’ in Guasti et al. (2023), and more broadly as a subtype of ‘commission’ in e.g., Snyder (2007, 2011). Several of these errors fit the definition of multiple or extended exponence (Caballero & Harris, 2012: 165; see also Matthews, 1974) as a feature or feature bundle is realized more than once. Errors along the lines of multiple exponence have been reported in multiple areas in Slobin (1973, 1985) and Karmiloff-Smith (1981), among others, and for the causative domain specifically, multiple exponence errors have been found in child French (Bezinska et al., 2008; Bezinska, 2014; Martin et al., 2022; Hein et al., 2024), child Turkish (Aksu-Koç & Slobin, 1985), child Japanese (Yamakoshi et al., 2018), and child English (Lord, 1979). We will briefly report on the results for French causatives as an introduction to the relevant more general pattern.

French has a class of lexical causative verbs like *montrer* ‘show’ or *fermer* ‘close’ which encode a causative meaning component (1). Causatives of verbs of other classes, e.g. the unaccusative *tomber* ‘fall’, may be formed by embedding them under *faire* ‘make’ (2).


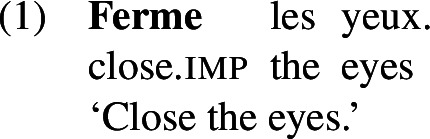



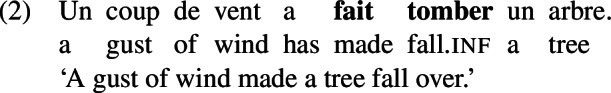


When a lexical causative verb is embedded under *faire*, the resulting construction is interpreted as a double causative in the adult language (1).


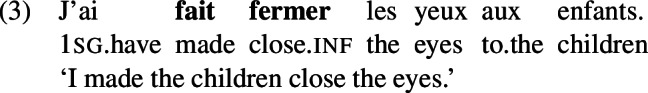
Children acquiring French have been found to sometimes produce lexical causatives embedded under *faire* with a meaning identical to the lexical causative alone (Bezinska et al., 2008; Bezinska, 2014). These superfluous productions of *faire* can be considered as multiple exponents of an underlying cause component, which is already expressed as part of the lexical causative itself.


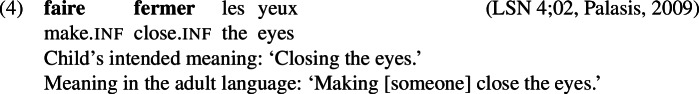


Importantly, Martin et al. (2023) show that children seem to be aware of the causative meaning of these verbs as well as of the causative contribution of periphrastic *faire*. They use both constructions in an adult-like manner alongside or even before producing errors of multiple exponence (1).


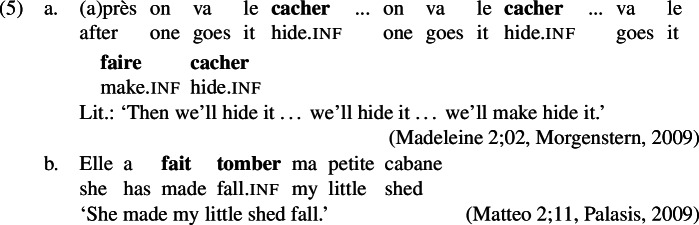


In a corpus study on French CHILDES, Martin et al. (2022) found that of all combinations of *faire* and an infinitive, roughly 10% were errors of multiple exponence.

This paper is concerned with the formation of past tense in child English. Commissive errors within the realm of English past tense marking are also known under the terms overregularization errors (Kuczaj, 1977, 1978; Stemberger, 1982; Marcus et al., 1992; Maratsos, 2000), doubling errors (Hattori, 2003) or overtensing errors (Stemberger, 2007). Overregularization occurs when an irregular verb’s stem is suffixed with the regular past tense marker *-ed*. The stem can either take the form that also appears in present tense, as shown in (1a), or it can appear in the portmanteau past tense form, which is often a suppletive or ab-/umlauted stem, as shown in (1b). In the latter case, as in the causative domain, a feature, here past tense, is marked twice, once by the stem allomorph and once by *-ed*, thereby constituting a case of multiple exponence.


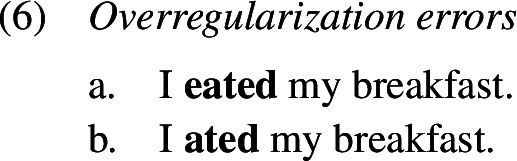


Overtensing, on the other hand, denotes cases of *do*-support where tense is marked both on *do* and also on the main verb, as in (1b). Like in the second type of overregularization error, past tense is realized more than once, albeit on two separate words. Overtensing errors have been documented to occur in non-emphatic contexts in both experimental and naturalistic settings.


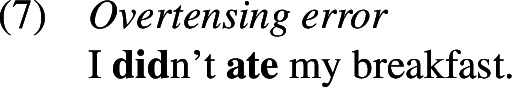
Overregularization and overtensing errors have figured in research on language acquisition since at least the late 70s (Kuczaj, 1977, 1978) and have been investigated from various angles including their frequency, acquisitional trajectory, and factors influencing them. Alongside multiple exponence errors, other types of errors have been documented (Kuczaj, 1977, 1978), where past tense is expressed only once but nevertheless spelled-out in a non-adult-like way.


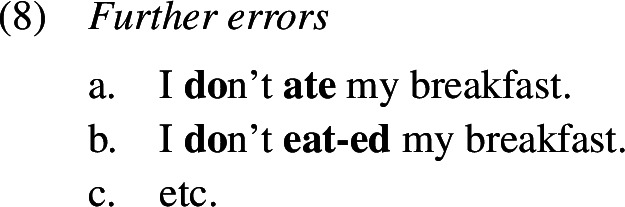
It is worth noting that errors of multiple exponence, such as (6b) and (7), are unexpected under the idea that children prefer a transparent one-to-one mapping between form and meaning and disprefer one-to-many mappings. The respective mappings between form and meaning for target forms of irregular verbs as well as both types of overregularization errors are visualized in (1). This (dis-)preference has been established in previous work on language acquisition and seems to be well-supported in various linguistic domains (Slobin, 1985; Brighton et al., 2005; van Hout, 2008; Guasti et al., 2023). Hence, an in-depth investigation of overregularization and overtensing errors should shed light on the tension between their existence and children’s preference for transparency.

Although various studies on corpora of child English have been conducted already, they either compare different error types of a set of verbs across limited corpora (Kuczaj, 1977; Marcus et al., 1992) or focus on one error type across different verbs in a larger number of corpora (Stemberger, 2007). Overregularization and overtensing errors have also mainly been studied independently of each other. As we are interested in comparing error frequencies and distributions of all error types within one and the same as comprehensive as possible data set, we conducted our own corpus study detailed below. This circumvents the problem that data from previously published corpus studies comes from different sources extracted by different methods across studies and error types. Further, it allows us to include corpora that were established in the past 20 years since the last queries on the phenomenon were carried out in late 2001 (Stemberger, 2007).

In order to be able to succinctly talk about the different types of errors, we establish here the terminology that we are going to use throughout the paper. Errors of the type *ate-d*, where past tense is expressed twice locally on one word, will be referred to as *redundant errors* (1c), while errors like *eat-ed*, where root information and past tense information unexpectedly occur distributed across two exponents within one word, will be called *distributive errors* (1b). As these errors occur locally on a single word, we will unify them under the term *local errors*.


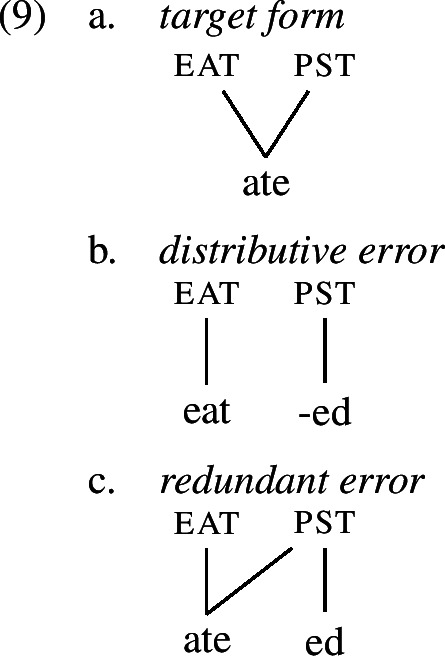
Tense errors within a *do*-support construction are termed *periphrastic errors*. Within these we distinguish *do-periphrastic errors* where past tense is expressed solely on the lexical verb as in *don’t ate* from *did-periphrastic errors* where it is marked on both *do* and the lexical verb as in *didn’t ate*.

After presenting the results of our corpus study of past tense formation in child English (Sect. 2), we will develop an account that derives all types of past tense errors, including errors of multiple exponence (Sect. 4), followed by an extension to cover multiple exponence errors in the causative domain. As past tense formation arguably involves some kind of head movement, our theory is based on the most recent and most comprehensive account thereof, i.e. *Generalized Head Movement* (Arregi & Pietraszko, 2021), which we introduce in Sect. 3. Finally, we provide some ideas on the distribution and frequency of the different types of errors in Sect. 5, before we conclude in Sect. 6.

## English past tense formation

In this section, we present our CHILDES corpus results on past tense formation in English, starting with the methodology and the overall results in Sect. 2.1. For each error type we provide examples, focusing on redundant errors in Sect. 2.2, periphrastic errors in Sect. 2.3, and distributive errors in Sect. 2.4.

### Corpus study

We targeted all British and North American English-language corpora (excluding African-American English) of typically developing children aged 1;01 to 15;11 available through the CHILDES database (MacWhinney, 2000) (as of July 2022). First, we determined the 100 most frequent verbs in the English-language CHILDES corpora through Sketch Engine’s wordlist function. Of the 44 irregular verbs in the list we excluded six from our investigation because their present and past forms are homographs (*cut, read, let, put, fit, hit*). Using the CLAN software’s ‘kwal’ command we then ran a query for past tense forms of 37 of the 38 remaining verbs, including distributive and redundant error forms in various orthographic variants.^1^ The corpora are listed in Table 1 and the selection of verbs is presented in Table 2.

**Table 1:** Summary of all corpora searched

North American English corpora	British English corpora
Bates (Bates et al., 1991); Bernstein (Bernstein, 1984); Bliss (Bliss, 1988); Bloom (Bloom, 1970); Braunwald (Braunwald, 1971); Brown (Brown, 1974); Clark (Clark, 1978); Demetras (Demetras, 1986); Ellis Weismer (Weismer et al., 2013); Evans (MacWhinney, 2000); Feldman (Feldman, 1998); Garvey (Garvey, 1979); Gelman (Gelman et al., 1998, 2004); Gillam (Gillam & Pearson, 2004); Gleason (Gleason, 1980); Hall (Hall William & Nottenburg, 1981); Higginson (Higginson, 1985); HSLLD (Dickinson & Tabors, 2001); Kuczaj (Kuczaj, 1977); MacWhinney (MacWhinney, 2000); McCune (McCune, 1995); McMillan (MacWhinney, 2000); Morisset (Morisset et al., 1995); New England (Ninio et al., 1994); Newman Ratner (Newman et al., 2016); Nicholas (Nicholas & Geers, 1997); POLER (Berl et al., 2005); Post (Post, 1992); Rondal (Rondal, 1976); Sachs (Sachs, 1983); Sawyer (Sawyer, 1997); Sprott (Sprott, 1992); Suppes (Suppes, 1974); Tardif (MacWhinney, 2000); Valian (Valian, 1991); Van Houten (Van Houten, 1986); Van Kleeck (MacWhinney, 2000); Warren (Warren-Leubecker, 1982); Weist (Weist et al., 2009)	Belfast (Henry, 1995); Conti (Conti-Ramsden & Dykins, 1991); Cruttenden (Cruttenden, 1978); Edinburgh (Ota et al., 2018); Fletcher (Fletcher & Garman, 1988); Forrester (Forrester, 2002); Gathercole/Burns (Gathercole, 1986); Howe (Howe, 1981); Kelly Quigley (Kelly et al., 2020); Lara (Rowland, 2007); Manchester (Theakston et al., 2001); MPI-EVA-Manchester (Lieven et al., 2009); Sekali (Beaupoil-Hourdel, 2015); Smith (Smith, 1973); Thomas (Lieven et al., 2009); Tommerdahl (Tommerdahl & Kilpatrick, 2014); Wells (Wells, 1981)

**Table 2:** Table of irregular verbs and the respective error forms searched

verb	Target	Distributive	Redundant	Frequency ranking
be	was, were	beed, amed, ared, ised	wased, wered	1
break	broke	breaked	broked	43
bring	brought	bringed	broughted	47
build	built	builded	builted	80
catch	caught	catched	caughted	75
come	came	comed	camed	10
do	did	doed, doesed	didded	2
drink	drank	drinked	dranked	72
drive	drove	drived	droved	86
eat	ate	eated	ated	18
fall	fell	falled	felled	29
feel	felt	feeled	felted	76
find	found	finded	founded	26
forget	forgot	forgetted	forgotted	89
get	got	getted	gotted	5
give	gave	gived	gaved	25
go	went	goed, goesed	wented	4
have	had	haved, hased	haded	3
hide	hid	hided	hidded	97
hold	held	holded	helded	39
keep	kept	keeped	kepted	48
know	knew	knowed	knewed	9
leave	left	leaved	lefted	37
lose	lost	losed	losted	92
make	made	maked	maded	15
mean	meant	meaned	meanted	40
run	ran	runned	ranned	53
see	saw	seed	sawed	7
sing	sang	singed	sanged	79
sit	sat	sitted	satted	24
sleep	slept	sleeped	slepted	57
stand	stood	standed	stooded	69
take	took	taked	tooked	19
tell	told	telled	tolded	21
think	thought	thinked	thoughted	11
throw	threw	throwed	threwed	51
wear	wore	weared	wored	78

Using a Python script, the hits were extracted into an Excel spreadsheet and automatically annotated for error type, TAR(get), DIS(tributive), and RED(undant), based on the CLAN command’s keyword output. In a second step, a further Python script and regular expressions were used to (i) check for error forms hidden in transcribers’ annotations (that the CLAN command ignored), (ii) reduce multiple hits for the same form within a single utterance if they involve retracings, and (iii) exclude participles of verbs where the participle is syncretic with the simple past tense form. The results were spot-checked for correctness. Furthermore, the script identified past tense forms that occurred as the complement of a form of *do*. These then underwent a complete manual validation. In total, this resulted in 103,590 tokens of past tense forms of these irregular verbs of which 100,674 could be classified as target-like, i.e. correct past tense forms. The remaining 2,916 occurrences resulted in an overall error rate of 2.81%; among these we found 382 redundant errors, 1,771 distributive errors, 416 tense errors in *do*-support constructions and 347 other errors not fitting the classification. Among the latter are mainly cases of past tense forms with 3rd singular agreement marker *-s* and present tense forms used for past tense meaning (as evidenced by the transcribers’ annotations in square brackets).

**Fig. 1:**
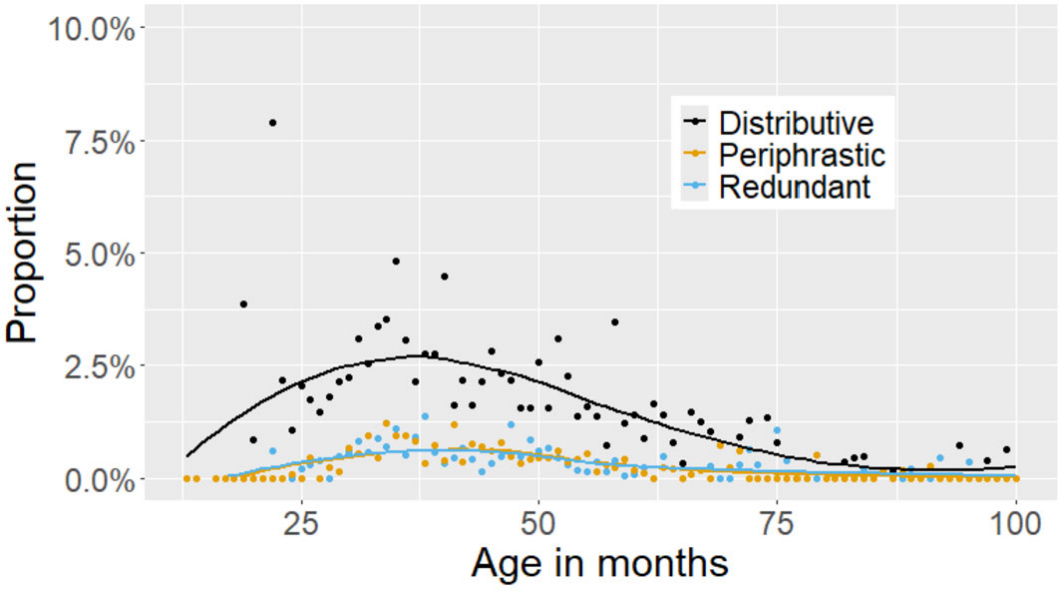
Error rates from 13 to 100 months (colour figure online)

The error proportions are given in Table 3. While the error rates are overall very low, they vary across ages, and thus cannot be a result of noise in the data. Figure 1 shows the distribution of error rates from 13 to 100 months.^2^ Errors are concentrated within a certain age span and then decline over time, as expected for errors which emerge and fade during the course of acquisition.

**Table 3:** Overall error counts across all ages

Type	*N*	%
target	100,674	97.19
distributive	1,771	1.71
redundant	382	0.37
periphrastic	416	0.40
did	365	0.35
do	51	0.05
other	347	0.33
total non-target	2,916	2.81

### Redundant errors

Starting with the least frequent error types, examples of redundant errors from the corpora are given in (1). As was the case in French causatives, we find that children must be aware both of the past tense meaning of portmanteau forms as well as of the past tense conveying meaning of *-ed* as they correctly use both alongside and before production of redundant errors, shown in (11) and (12). This is not surprising since it has been noted in the literature that overregularization (i.e. redundant) errors only start occurring after the child has acquired the relevant rule, which in our case is the expression of past tense by affixing *-ed*.^3^


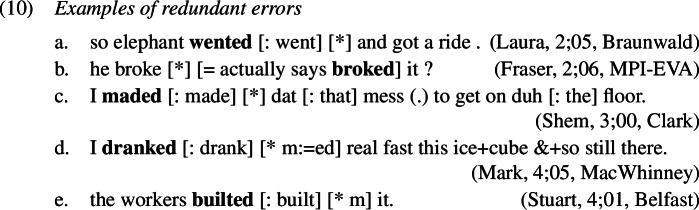



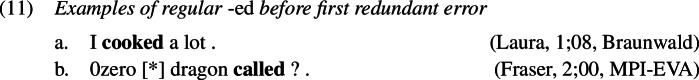



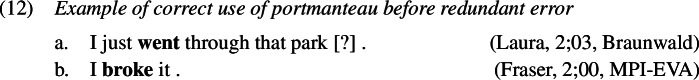
The distribution of redundant errors over age is provided in blue in Fig. 1. We find an increase and decrease in error frequency over an age span of about 90 months starting from 13 months and reaching zero at roughly 100 months of age. In fact, the latest redundant error in our sample is produced by a 95 months-old child. Limiting ourselves to data produced up to and including 100 months of age (93,356 tokens), the redundant error rate in this age span marginally increases to 0.41%. The error rate for redundant errors peaks between 35 and 45 months at roughly 0.7%.

### Periphrastic errors

A similarly frequent error type are periphrastic errors; a few examples are given below of both the *did*- (1a–d) and *do*-types (1e–g). Again, we find that *do*-support is used correctly alongside and before the production of periphrastic errors (14), that is, the child produces an inflected form of *do* and the infinitive of the lexical verb. Manual inspection of the local discourse contexts for these periphrastic errors confirms that the vast majority of them (397 or 95%) appear either in a question or a negation context, that is, they are most probably not cases of emphatic *do*-support or verum focus on an expected simplex past tense verb form.


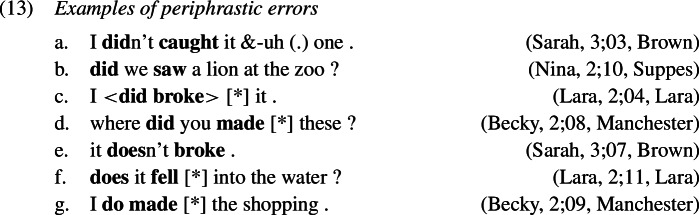



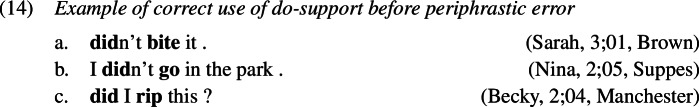


Again we find that the error rate approaches zero at around 100 months of age. Only 5 errors are attested beyond the age of 100 months, which brings its error rate between 0–100 months to 0.44%. The error rate peaks between 35–45 months at circa 0.7%. It should be mentioned that these rates are calculated based on all past tense contexts. Since only a subset of these are also *do*-support contexts (e.g. questions, negations) the actual error rate for periphrastic errors based only on contexts where we actually expect *do*-periphrasis in the first place is likely much higher.^4^

Within periphrastic errors we find that the *did*-type, where past tense is marked on both *do* and the lexical verb, is roughly 7 times more frequent than the *do*-type, where past is expressed on the lexical verb only.

### Distributive errors

Errors of the distributive type are by far the most frequent, being 4 to 5 times more frequent than the other two error types under investigation here.^5^ They differ from the other types in that they involve no redundancy. Examples from the corpora are given in (1).


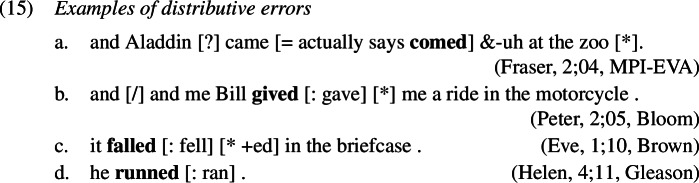


As with the other two types, the same children use the correct portmanteau form alongside and before they produce distributive errors.


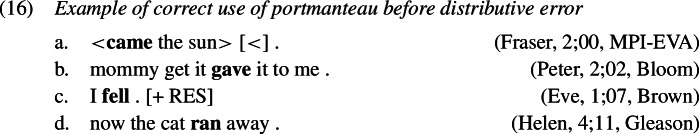


The distributive error rate peaks – slightly earlier than the others – between 30 and 40 months at roughly 2%. Nonetheless, this type of error also fades out at around 100 months of age, with only 5 errors occurring above that age.

A similar pattern in the relative frequency of different error types is found in the domain of negative indefinites in German and Dutch by Driemel et al. (2023). In these languages, the two semantic units negation and existential are usually expressed by a portmanteau form, the negative indefinite determiner *k-ein*/*g-een*, similar to how the two units root and past tense are expressed by a portmanteau stem form for some English verbs, e.g. *ate*. Based on a corpus study, Driemel et al. (2023, p. 38, fn. 26) report that children produce distributive errors of the form *nicht ein*/*niet een* ‘not a’, which correspond to errors of the type *eat-ed* in the current study, and redundant errors, where a single semantic negation is seemingly expressed twice, like *nicht kein*/*niet geen* ‘not no’, which correspond to errors of the type *ate-d* here. Interestingly, the distributive errors in this domain are also more frequent (2.5% in German, 5.3% in Dutch) than the redundant ones (1.6% in German, 0.7% in Dutch). This parallel might indicate a more general, domain-independent force behind those error patterns, which we argue to be found in the children’s bias for transparent one-to-one mappings between underlying concepts and overt surface forms. We will discuss how this bias plays out in the domain of past tense in English in Sect. 4.1.

In the remainder of the paper, we will explore which part of the grammar children struggle with in order to derive the observed non-adult like utterances. Past tense formation in the target grammar is analyzed within the framework of Distributed Morphology (Halle & Marantz, 1993, 1994), where verbal features end up in a local configuration due to Generalized Head Movement (Arregi & Pietraszko, 2021). In a nutshell, children fail to produce the right target forms from time to time because they sometimes ignore secondary features of the relevant Vocabulary Items due to an inherent bias for one-to-one mappings between form and meaning/features.

## Generalized Head Movement (GenHM)

Redundant and distributive errors are local errors, which can be fairly straightforwardly derived in approaches that employ classical head movement to unite the lexical verb with tense information (Koopman, 1984; Travis, 1984; Baker, 1985). However, such approaches have difficulty accounting for periphrastic errors, in which the auxiliary verb and the lexical verb both exhibit tense marking in a non-local configuration. Tense information must therefore be available at two independent sites in the structure. To capture this, we adopt the theory of Generalized Head Movement (henceforth GenHM) as proposed in Arregi and Pietraszko (2021). GenHM is an explicitly formalized account of head displacement which captures all phenomena commonly accounted for by, and additionally also patterns that have proved problematic for, classical head movement. Its main feature for the purposes of this paper, and the reason for adopting it, however, is the way *do*-support is treated as a defective copy of the lexical-verb-plus-tense complex. In contrast to competing approaches to *do*-support (e.g. Schütze, 2004; Bjorkman, 2011; Thoms, 2012) it thereby provides us with a straightforward handle on periphrastic errors as we will detail in Sect. 4.2. We also adopt it in our analysis of the local errors in Sect. 4.1 for reasons of consistency. It should be noted, however, that the explanation for local errors we present there straightforwardly transfers to approaches that employ classical head movement. Before we present the analysis of the various error types in Sect. 4, we therefore first introduce GenHM in Sect. 3.1 and in particular its treatment of *do*-support in Sect. 3.2.

### Complex verb formation in GenHM

GenHM unifies upwards and downwards displacements of heads in one distinct operation. The operation is triggered by a feature [hm] on the higher head. Arregi and Pietraszko (2021) assume that features of a head that are relevant to spellout, i.e. those that underlie morphological distinctions, constitute a set that is the value of a larger [M] feature. These bundled *morphological features* contrast with *syntactic features*, which are involved in structure-building and are hosted on the syntactic heads directly, i.e. not bundled under a larger feature. Abbreviating the value of M (that is, the set of morphological features) on a given Head H as H_m_, GenHM is defined in (1).


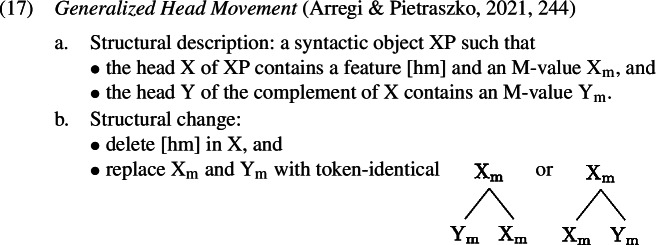
The result of an application of GenHM is therefore an object with the internal structure of a complex head (though only involving the M-values of the heads). That is, while classical head movement creates complex head structures by adjoining the moving syntactic head to the attracting one (Koopman, 1984; Travis, 1984; Baker, 1985), GenHM creates complex M-values, where the spellout-relevant features that are the value of the lower head’s M-feature are arranged in a hierarchical structure with the spellout-relevant features that are the value of the higher head’s M-feature. This hierarchical structure is then shared between all targeted heads as the common value of these heads’ M-features, as depicted fully in (1a) and in abbreviated form in (1b). The syntactic heads themselves never undergo actual displacement; they merely share the same complex M-value (cf. Agree as feature-sharing; Pollard & Sag, 1994; Brody, 1997; Frampton & Gutmann, 2000; Pesetsky & Torrego, 2007). Thus, despite the appearance, the result of GenHM, i.e. the complex structure headed by Y_m_ in (1a), is not a complex syntactic head but rather a set of hierarchically organised features. As we will detail momentarily, however, for the purposes of Vocabulary Insertion this complex feature structure behaves like a complex syntactic head created by classical head movement.^6^


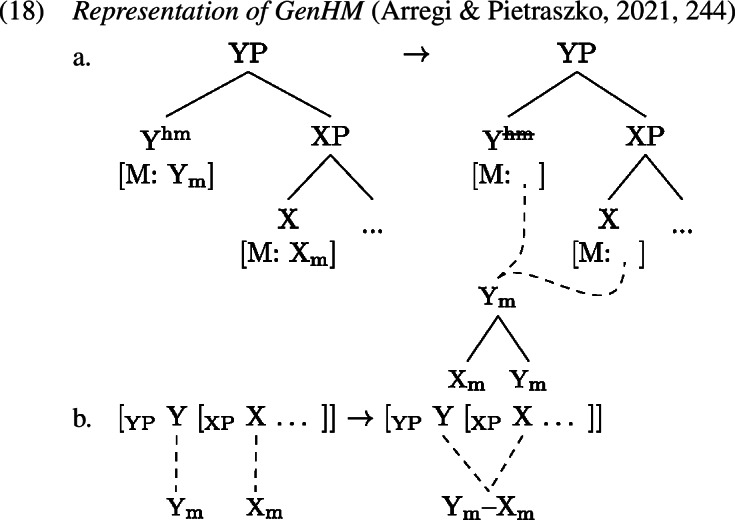
In a realizational morphological framework such as Distributed Morphology (Halle & Marantz, 1993, 1994) it is usually the syntactic terminals, i.e. the individual heads, that are targeted for Vocabulary Insertion which consists in rewriting the relevant morphosyntactic features with phonological material, i.e. the actual exponents (Noyer, 1997; Bobaljik, 2000; Embick, 2015). However, since after application of GenHM, all features of the single heads that are relevant for this process are organized in a hierarchical complex (0a), Arregi and Pietraszko (2021) suggest that Vocabulary Insertion actually targets the terminals of this complex structure, called morphological terminals, in a bottom-up fashion starting with the most deeply embedded ones (Bobaljik, 2000; Embick, 2010). The actual locus of pronunciation of the shared M-value is regulated at PF by the following principle, where a *head chain* refers to all heads that share a single M-value and where strength is encoded by a diacritic feature ‘*’ that is part of the lexical properties of some syntactic terminals.


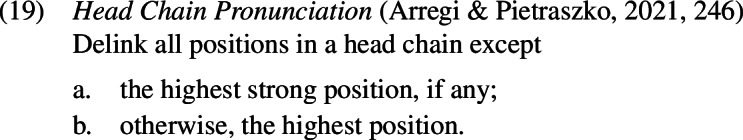
This principle models cross-linguistic variation, along with the assignment of strength to the heads in question. One well known cross-linguistic difference between English and French is the position of the adverb, which signals the presence of verb displacement in French in contrast to English (Pollock, 1989), see (20) and (21).


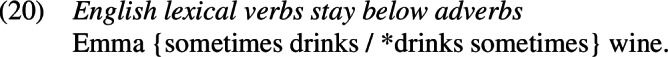



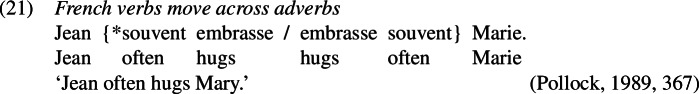
This classic difference between English and French verb movement is modelled in GenHM as a difference in strength on lexical verbs such that they are strong in English, effecting pronunciation of the T–V head chain in the V position (by clause 19a), but weak in French, leading to pronunciation in the T position (by clause 19b). As the delinked (✗) structures in (22) and (23) indicate, this explains the placement of the verb relative to the adverb whose position is fixed at the VP-level (Arregi & Pietraszko, 2021, 247).^7^


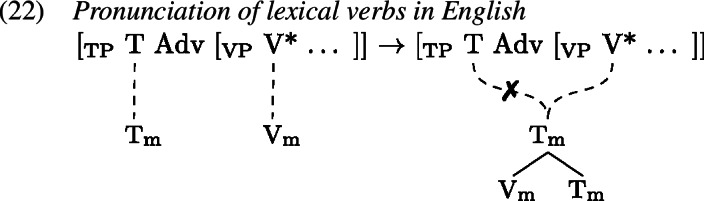



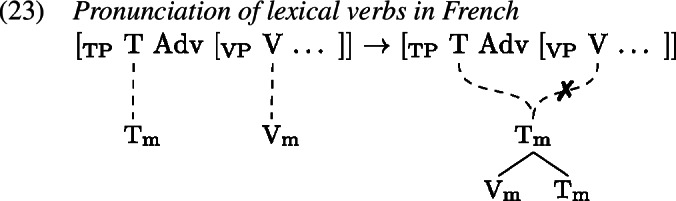
GenHM can apply iteratively, creating longer head chains with ever more complex M-values.

### GenHM and *do*-support

Certain environments seem to block the formation of a complex verb; one is negation and the other is subject-auxiliary-inversion, e.g., in the case of *wh*-questions.


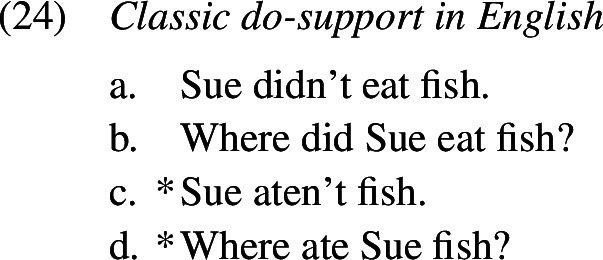
In such environments, we find that tense information on T and the lexical verb in V are pronounced separately. Arregi and Pietraszko (2021) argue that T and V nonetheless uniformly undergo GenHM in those contexts sharing a single complex M-value, just as they do in non-emphatic positive declarative sentences (cf. (22)), but that the resulting head chain is subject to a splitting operation. This operation is triggered by [+P] marked specifiers that intervene between the syntactic heads in a head chain. It splits the head chain into two such that each of the resulting head chains is linked to a type-identical copy of the original head chain’s M-value.


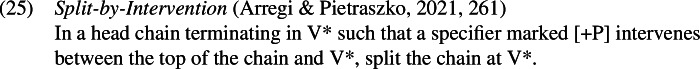
Assuming that sentential negation is introduced as the specifier of a silent Σ head which is itself part of the head chain between T and V, (0) induces a split at V*. Similarly, the subject in SpecTP triggers a split in the head chain relating C, T and V in Subject-Auxiliary-Inversion contexts. The split effectively copies the complex M-value structure leading to two identical M-values, one linked to the M-features of the higher heads T and Σ in (1), C and T in (2), and another linked to the M-feature of V* in both (1) and (2).


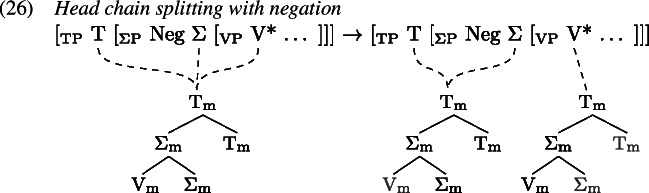



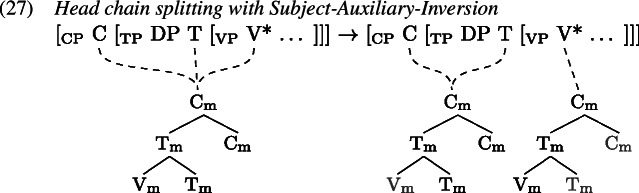
Having split the chain, we now are facing a situation where some M-values within the complex structure created by GenHM are no longer associated with the syntactic terminals that they originated from (marked in ). In (27) for example, $\mathrm{V}_{\text{m}}$ in the higher M-value is not associated with V*, while in the lower M-value $\mathrm{T}_{\text{m}}$ and $\mathrm{C}_{\text{m}}$ are not associated with T and C. A process of Orphan Assignment then assigns the feature [O] to those morphological terminals in an M-value structure that are no longer associated with their respective syntactic terminals.


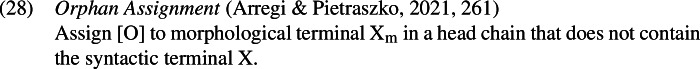
This [O] feature is referenced by Vocabulary Insertion such that $\mathrm{V}_{\text{m}}^{[\text{O}]}$ will always be realized by *do* in the highest position of the higher head chain, i.e. T or C (by clause b of *Head Chain Pronunciation* in 19). Importantly for our purposes, an [O] feature on $\mathrm{T}_{\text{m}}$ triggers obliteration of $\mathrm{T}_{\text{m}}$. The lower head chain will therefore be pronounced as an infinitive form of the verb in the position of V*. Delinking (✗) according to (19) applies after Orphan Assignment as part of the linearization algorithm.


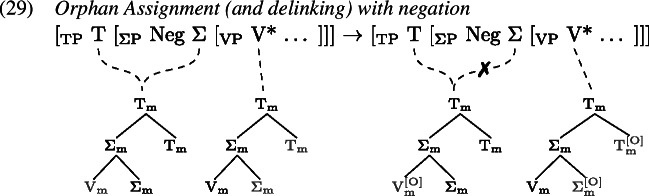



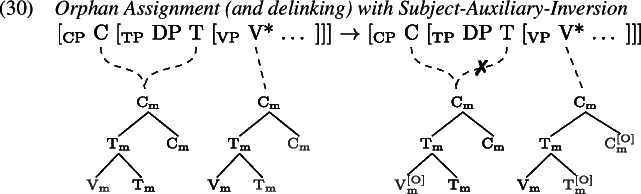
Generalized Head Movement thus allows us to retain that (i) *do* is a realization of some verbal head (Embick & Noyer, 2001; Thoms, 2012) and that (ii) it appears to occupy the position of T (Chomsky, 1957, 1995; Lasnik, 2000) in spite of the fact that V and T seemingly do not interact via Head Movement or Lowering in environments triggering *do*-support. More important for the analysis of children’s periphrastic errors, however, is that the tense information $\mathrm{T}_{ \text{m}}$ is underlyingly present both on what is being realized as a form of *do* and on what is realized as a non-finite form of the lexical verb.

## Deriving children’s errors

With GenHM in place, we can now turn to the question of how this can account for the variety of children’s errors that we presented in Sect. 2. We first focus on the derivation of the English past tense data, motivating our account of secondary feature negligence as the source for redundant and distributive errors in Sect. 4.1, which ultimately also predicts the occurrence of omissive errors. Periphrastic errors are specific to the domain of English past tense and will be accounted for in Sect. 4.2, based on the assumptions of GenHM. For illustrative purposes, we focus on errors with the verb *eat*, as it has figured prominently in the literature. However, the rationale applies to irregular verbs generally. In Sect. 4.3, we briefly show how our account of secondary feature negligence extends to French causative errors of multiple exponence.

### Secondary feature negligence results in distributive, redundant and omissive errors

A redundant error in the English past tense occurs when the past tense allomorph of an irregular verb is suffixed with the regular past tense marker *-ed* such that in effect the tense information is realized by two distinct exponents, as in *ate-d*. We first map out how the target past tense forms are derived, after which we turn to the derivation of the redundant errors.

Following the GenHM approach laid out above, there is a head chain in English verb forms consisting of (at least) the syntactic terminals V and T which share the complex M-value $\mathrm{V}_{\text{m}}$–$\mathrm{T}_{\text{m}}$ as shown in (1).


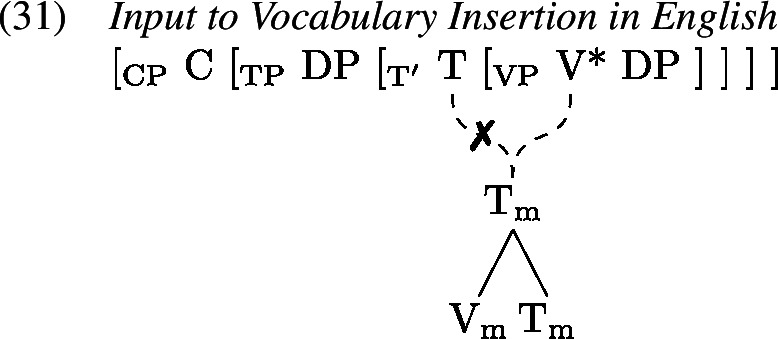
It is this complex M-value that is subject to Vocabulary Insertion starting from the most deeply embedded morphological terminal, i.e. $\mathrm{V}_{ \text{m}}$ here. For a target regular past tense verb form, both morphological terminals are simply realized separately each by their own VI. The VI inserted into $\mathrm{V}_{\text{m}}$ would be a regular verb stem (probably realizing a root of some sort) while the VI realizing $\mathrm{T}_{\text{m}}$ is the regular past tense suffix *-ed* (1).


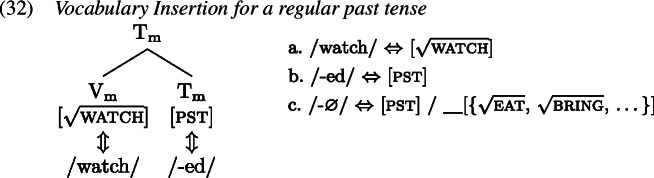
For target irregular verbs, there are then two possibilities to ensure the insertion of a special past tense form. As a first option, one can treat the irregular form as a proper portmanteau form realizing the features of both $\mathrm{T}_{\text{m}}$ and $\mathrm{V}_{\text{m}}$ equally. In this case, $\mathrm{T}_{ \text{m}}$ and $\mathrm{V}_{\text{m}}$ would have to be fused into a single morphological terminal prior to Vocabulary Insertion because it can only target one node at a time. Insertion of the portmanteau into either $\mathrm{V}_{\text{m}}$ or $\mathrm{T}_{ \text{m}}$ is precluded by the Subset Principle as the features of the portmanteau do not constitute a subset of the features of either one of the morphological terminals separately.


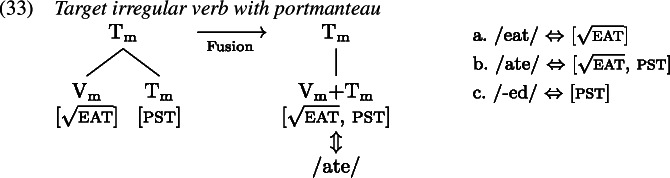
The second option is to treat the irregular form as a contextual allomorph of the regular stem exponing the root in the context of past tense. That is, while the irregular form primarily realizes the verbal root, it bears a secondary past tense feature that must be present on a terminal node in the local environment of $\mathrm{V}_{\text{m}}$, i.e. on $\mathrm{T}_{\text{m}}$ (Carstairs, 1987; Noyer, 1997). In this case, there must be a zero VI in the lexicon that realizes past tense in $\mathrm{T}_{\text{m}}$ just when the root is realized by an irregular verb form. This can be achieved by specifying the zero exponent’s secondary feature as a list of roots (Embick, 2003). By virtue of this secondary feature, the zero exponent is more specific than the regular *-ed* exponent and therefore takes precedence. A list of relevant Vocabulary Items is given in (34), while the process of Vocabulary Insertion is shown in (35).


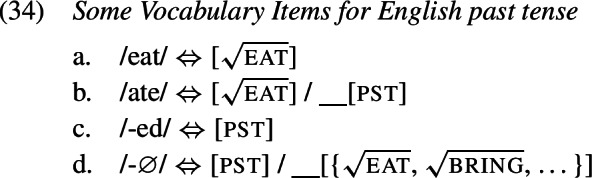



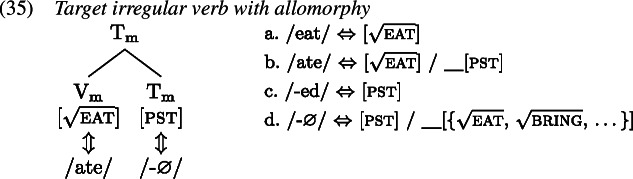
We adopt the latter option in this paper for two main reasons (cf. Bobaljik, 2000; Trommer, 2010; Fenger, 2018 for arguments for contextual allomorphy in the domain of person portmanteaux). First, the notion of Fusion as necessitated by a proper portmanteau approach to irregular verbs is problematic. In particular, it is unclear how its application is regulated and/or triggered, i.e. why it only takes place with irregular verbs and is blocked from applying to regular ones (though see Siddiqi, 2006 for a proposal). The second reason is that children’s redundant errors are near identical to instances of multiple or extended exponence in adult languages (Matthews, 1974). Within Distributed Morphology these are typically modelled via secondary features (Noyer, 1992), where the apparent secondary exponent of a feature’s lexical specification contains that feature as a secondary feature entry.

In light of the fact that children produce target forms before and alongside commissive errors we can assume that the children have acquired full lexical entries for the verb forms and inflectional affixes, including secondary feature specifications. Building on Hein et al. (2024), we contend that the production of a local error, e.g. an occurrence of multiple exponence of past tense, is the result of the child’s sporadic flouting of specificity, in particular when secondary features are involved. This proposal of secondary feature negligence is formulated as in (1).


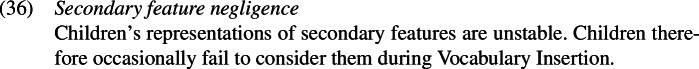
Negligence of secondary features essentially leaves the relevant Vocabulary Items specified for a single meaning component. For example, when the Vocabulary Item for /ate/ (-1b) is stripped of the secondary feature [pst] only the primary feature [] remains. This is true for all Vocabulary Items involved in the errors discussed in this paper. Negligence of secondary features therefore directly turns a one-to-many mapping between form and meaning (i.e. features) into a one-to-one mapping which is in line with children’s preferences as mentioned in Sect. 1.

To see how negligence of secondary features leads to a redundant error, let us consider the derivation of *ate-d*. A range of examples for redundant *ate-d* is given in (1).


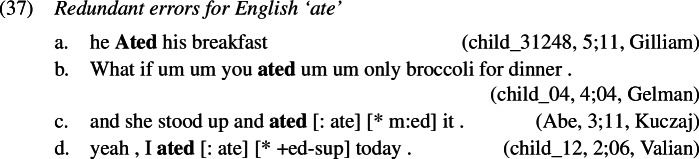
The relevant Vocabulary Items were given in (34), where *eat* simply realizes the root  while the more specific allomorph *ate* realizes the root in the context of a past tense feature (on a different terminal in the local domain). Similarly, the regular realization of the feature [pst] is *-ed* while its more specific zero allomorph appears in the context of the listed roots.

As Vocabulary Insertion proceeds from the root outwards (Bobaljik, 2000; Kalin & Weisser, 2021) it first applies to $\mathrm{V}_{\text{m}}$. Both the default allomorph *eat* (1a) as well as the past tense variant *ate* (1b) are viable candidates. Among them, the more specific *ate* should be chosen based on its additional secondary feature. In a redundant error, the child manages to correctly select and insert *ate*. In the next cycle, Vocabulary Insertion targets $\mathrm{T}_{ \text{m}}$. Again, both available allomorphs, *-ed* (1c) and ∅ (1d), are compatible with the features in $\mathrm{T}_{ \text{m}}$ and its context. By virtue of its secondary feature list of roots, ∅ is expected to win the competition. However, the child fails to take into account ∅’s secondary features for calculation of specificity and inserts the default *-ed*, generating the output *ate-d*.


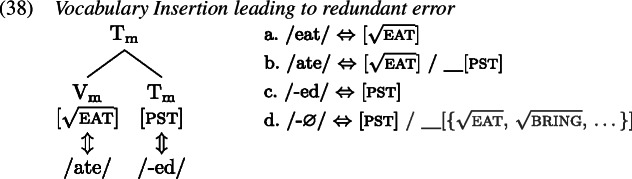
In summary, for a redundant error the child fails to respect specificity based on secondary features only when inserting into $\mathrm{T}_{\text{m}}$. Insertion into $\mathrm{V}_{\text{m}}$ takes place in an adult-like fashion.

This analysis of redundant errors implies that children may variably flout specificity for Vocabulary Insertion into some heads but not others within one and the same derivation. This variability can be exploited when accounting for other types of errors observed in children’s past tense productions, such as distributive errors. Some examples of this type of error are given in (1).


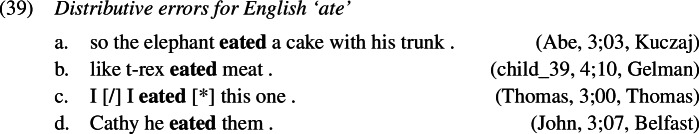
With these errors, the child can be taken to ignore secondary feature specificity for insertion into both $\mathrm{T}_{ \text{m}}$ and $\mathrm{V}_{\text{m}}$. Thus, the less specific allomorph *eat* (1a) is erroneously chosen to realize $\mathrm{V}_{ \text{m}}$, while the less specific allomorph *-ed* (1c) is again mistakenly picked for insertion into $\mathrm{T}_{\text{m}}$ in the following cycle. This flouting of specificity in both terminals leads to the distributive production *eat-ed*.


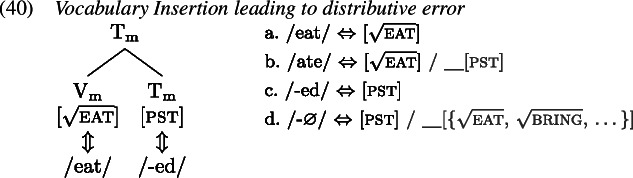
With two morphological terminals, there are two more spell-out possibilities. One is the case where the child fully respects specificity in both locations. This, of course, results in an adult-like correct past tense production as in (35). A third error occurs when the child ignores secondary features for insertion into the root $\mathrm{V}_{ \text{m}}$ but not for insertion into $\mathrm{T}_{\text{m}}$. In this case, the stem allomorph would come out as *eat* (1a), while the past tense marker would occur as ∅ (1d). The overall production is then indistinguishable from the present tense form of the verb as no overt past tense marking is present; see (1). We can therefore classify this absence of past tense marking in a past tense environment as belonging to the class of omission errors (Matthews & Theakston, 2006).


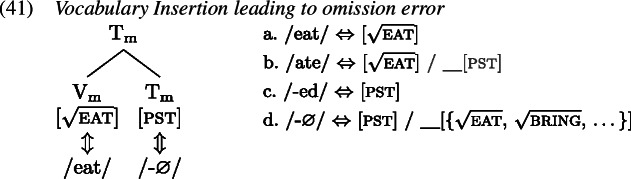
In summary, we can attribute the range of observed errors in the past tense productions to a single mistake: neglecting specificity based on secondary features. This mistake can be made in (at least) two different locations, $\mathrm{V}_{\text{m}}$ and $\mathrm{T}_{\text{m}}$. This gives rise to four different patterns associated with three distinct error types and the target form, shown in Table 4.

**Table 4:** Typology of (local) past tense errors

$\mathrm{V}_{\text{m}}$	$\mathrm{T}_{\text{m}}$	Error location	Error type
*ate*	*-*∅	none	target
*eat*	*-*∅	$\mathrm{V}_{\text{m}}$	omissive
*eat*	*-ed*	$\mathrm{V}_{\text{m}}$ & $\mathrm{T}_{\text{m}}$	distributive
*ate*	*-ed*	$\mathrm{T}_{\text{m}}$	redundant

With regard to the overall error rates, it might seem counterintuitive that distributive errors, where the child makes mistakes on both terminals, are more frequent than redundant errors, where she only makes one mistake. One would expect the probability of making one mistake to be higher than that of making one mistake and then another one immediately after the first. We will come back to this issue in Sect. 5.

Overall, we believe that secondary feature negligence is driven by the children’s preference for one-to-one mappings between form and meaning when acquiring the target language (Slobin, 1985; Brighton et al., 2005; van Hout, 2008; Guasti et al., 2023). To illustrate our point we show the mapping from form to meaning for the past tense forms of *eat* in (42).^8^


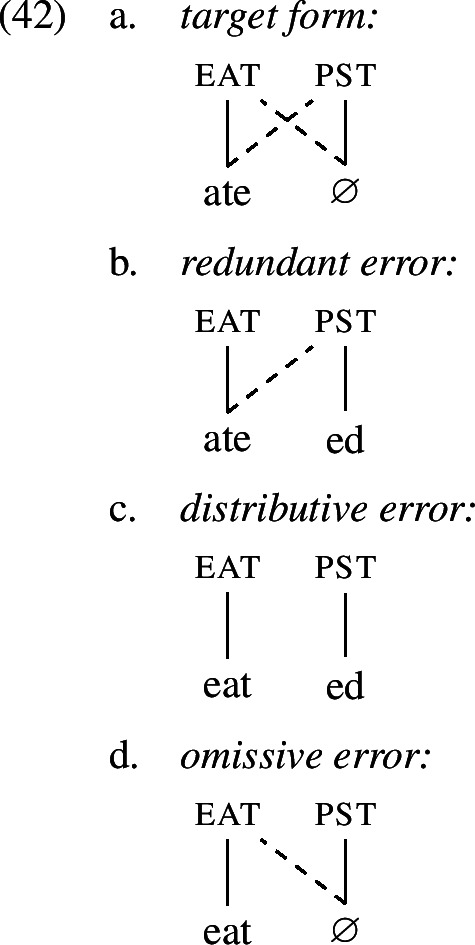


The Vocabulary entries in (34) indicate that in order to build the target form (42a) both eat and pst need to be spelled out by VIs that make reference not only to the primary feature they encode (solid line), but also to a secondary feature via a contextual restriction (dashed line). The children’s errors result from breaking up this double association in one way or another. To produce a distributive error (42c), the child chooses a fully transparent one-to-one mapping, ignoring secondary features in both places, so that *eat* maps to eat and *-ed* maps to pst. This drive for transparency is also present in the production of redundant (42b) and omissive errors (42c), where at least one mapping is simplified to a one-to-one association. Thus, secondary feature negligence is a symptom of a more general acquisition principle, a requirement for transparent mappings between form and meaning, which is assumed to be at play across several linguistic domains.

A reviewer asks whether this negligence of secondary features predicts any unattested error types. As it basically leads to featural identity between /eat/ ⇔ [] and /ate/ ⇔ [] as well as between /-ed/ ⇔ [pst] and /-∅/ ⇔ [pst] we should expect /ate/ to falsely occur in contexts where /eat/ is expected, for example in the present tense or in place of infinitives after e.g. *to* or *will*. Likewise, we should find /-∅/ as a past tense marker with regular verbs that usually show the overt past tense exponent /-ed/. In fact, these types of errors are attested in our data. Some relevant examples that we extracted incidentally in our corpus search for past tense forms are given in (1), where the annotator indicates a missing /-ed/ with “[* 0ed]”.^9^


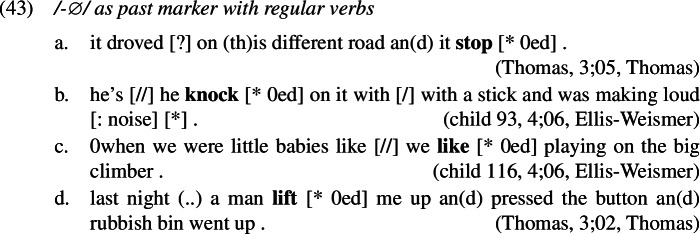
It is worth noting that these errors also fall under the category of so-called root infinitives (see e.g. Harris & Wexler, 1996; Legate & Yang, 2007; Phillips, 2010, among many others), which might have a different source than our omissive errors (see Wexler, 1998; Guasti, 2002 for overview and discussion of different theories).

As for occurrences of past tense stem forms in a context where no past tense feature is present and hence a present tense stem is expected, such as after a present tense modal or a future auxiliary or after *to*, these are also attested in our data. Examples are provided in (1).


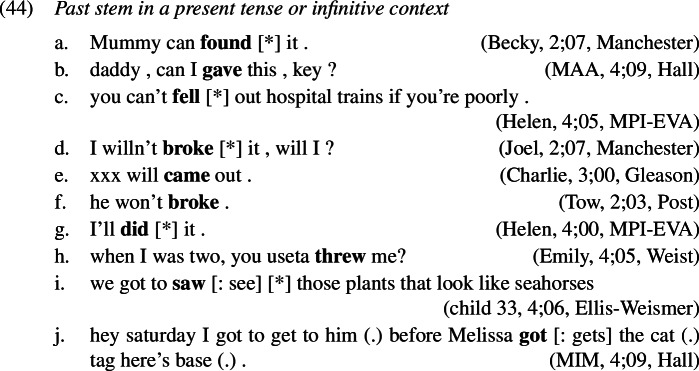
Thus, the predictions of secondary feature negligence pointed out by the reviewer are actually borne out.

Before we explore how our account of secondary feature negligence can be extended to the causative domain, we consider one more type of error which is specific to the domain of English past tense and where the theory of GenHM becomes a crucial component of our proposal.

### Periphrastic errors in the English past tense

In the domain of past tense marking in English, we find a further type of error that does not occur in other domains such as the causatives, for instance. As the English verbal system distributes the exponence of the lexical content of the verb and its inflectional content in certain environments known as *do*-support, it is possible that past tense is marked on both, the support element *do* and the lexical verb itself, resulting in what we have called a *did*-periphrastic error, another instance of multiple exponence. We also find *do*-periphrastic errors, where past tense is expressed on the lexical verb instead of the support verb *do*. A few illustrative examples are given in (1), within *do*-support environments including negation, *wh*-questions, and polar questions.


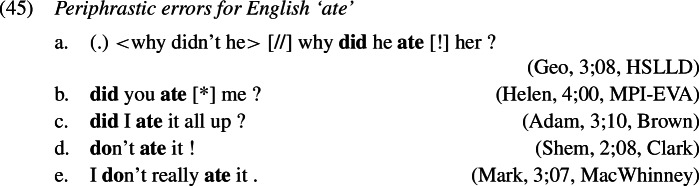
Recall from Sect. 3 that the derivation of *do*-support with GenHM involves an instance of head chain splitting at V*, such that there are two type-identical complex morphological values, one of which is associated with T and Σ/C, while the other one is associated with V*. In addition, in each M-value those morphological terminals that are no longer linked to their respective syntactic terminals have been marked as orphans ([O]). The input to Vocabulary Insertion is therefore slightly different in environments that involve *do*-support. We choose to demonstrate our account based on the *did*-periphrastic errors in (0). As these involve Subject-Auxiliary-Inversion, the chain split structure in (1) serves as the input to Vocabulary Insertion.


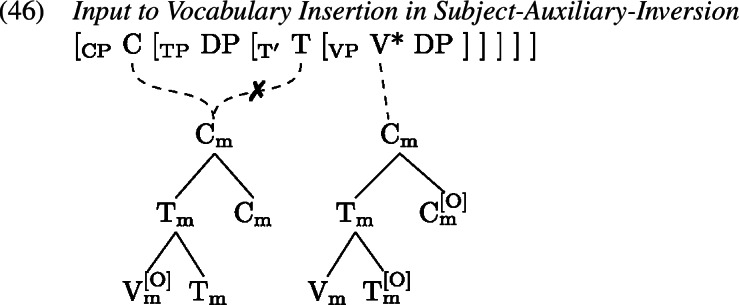
When these M-values undergo Vocabulary Insertion (1), the [O] feature plays an important role. In the adult language, an [O]-marked $\mathrm{V}_{ \text{m}}$ is invariably realized as a form of *do*, in this case *did* as $\mathrm{T}_{\text{m}}$ carries past tense information. In contrast, the [O] feature on $\mathrm{T}_{\text{m}}$ triggers obliteration (Arregi & Nevins, 2012) of the morphological terminal $\mathrm{T}_{\text{m}}$ (marked as ). Since all obliteration and impoverishment rules precede all Vocabulary Item insertions, root allomorphy is bled, and instead $\mathrm{V}_{\text{m}}$ is realized as *eat*. $\mathrm{C}_{\text{m}}$ is always realized by a ∅ exponent.


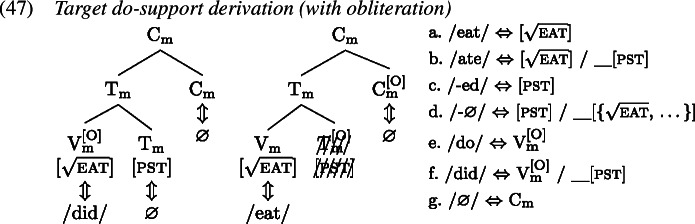
A *did*-periphrastic error then results if a child fails to properly obliterate $\mathrm{T}_{\text{m}}$, shown in (1). In that case, tense information can condition the choice of realization of $\mathrm{V}_{\text{m}}$, given that the local domain for contextual allomorphy is the whole complex M-value. In other words, a VI specified for secondary tense features, like *ate*, is compatible with $\mathrm{V}_{\text{m}}$ and more specific than its competitor *eat* and should therefore be inserted. $\mathrm{T}_{\text{m}}$ itself is realized by the most specific marker ∅. The M-value associated with the syntactic terminals T and C also contains tense information. When insertion targets $\mathrm{V}_{\text{m}}^{[\text{O}]}$, the past tense allomorph *did*, which comes with a secondary [pst] feature specification, wins over the default allomorph *do*. This results in a surface form where past tense is marked twice, once on the support element and once on the lexical verb. The only mistake that the child has made, however, is that they have failed to obliterate $\mathrm{T}_{\text{m}}$.


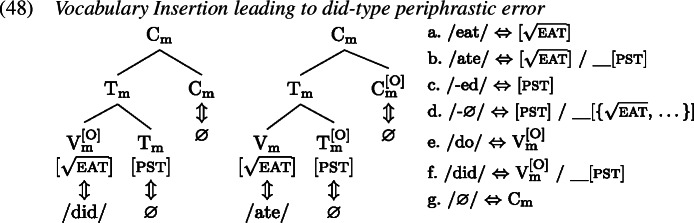
How does the failure to obliterate tense information tie in with the transparency principle (Slobin, 1985; van Hout, 2008) children follow when producing periphrastic errors? In a sense, obliteration creates opacity between underlying information and surface forms. It is a process that takes away information, before spell-out can make reference to it. So by the non-application of obliteration, children do indeed create more transparent one-to-one mappings. Interestingly, for *do*-support scenarios this bias towards transparency results in the multiple exponence of past tense information. This outcome is, however, entirely expected by GenHM given that *do*-support contexts in English are derived by head chain splitting, which ultimately results in the doubling of $\mathrm{T}_{\text{m}}$, as was shown in (46).

In addition to the failure to obliterate $\mathrm{T}_{\text{m}}$, a child can of course also make the abovementioned specificity errors. In such a case, a variety of error patterns is expected, including *do*-type periphrastic errors. An overview of these error types is given in Table 5. Focusing on the M-value linked to V* first, the child could fail to insert the most specific allomorph in $\mathrm{T}_{\text{m}}$ or in both $\mathrm{T}_{\text{m}}$ and $\mathrm{V}_{\text{m}}$. This would result in errors like *did … ated* and *did … eated*, respectively. If a specificity error is made on $\mathrm{V}_{\text{m}}$ only, the result would look just like a target production *did … eat* on the surface. Turning to the M-value associated with the T and Σ/C heads, specificity errors can likewise be made either on $\mathrm{T}_{\text{m}}$ or $\mathrm{V}_{\text{m}}$, or both. This would result in errors such as *didded … ate*, *do … ate* and *doed … ate*, respectively. If specificity errors are made on both M-values, a further set of errors results, namely those that consist of a combination of the aforementioned ones, i.e. *didded … ated*, *didded … eated*, *do … ated*, *do … eated*, *doed … ated* and *doed … eated*.

**Table 5:** Types of periphrastic errors (no obliteration of $\mathrm{T}_{\text{m}}^{[ \text{O}]}$)

High M-value		Low M-value		Secondary feature negligence		Total
$\mathrm{V}_{\text{m}}^{[\text{O}]}$	$\mathrm{T}_{\text{m}}$	$\mathrm{V}_{\text{m}}$	$\mathrm{T}_{\text{m}}^{[\text{O}]}$	Location high	Location low	
*did*	*-*∅	*ate*	*-*∅	none	none	356
*did*	*-*∅	*ate*	*-ed*	none	$\mathrm{T}_{\text{m}}^{[\text{O}]}$	1
*did*	*-*∅	*eat*	*-ed*	none	$\mathrm{V}_{\text{m}}$, $\mathrm{T}_{\text{m}}^{[\text{O}]}$	8
*did*	*-*∅	*eat*	*-*∅	none	$\mathrm{V}_{\text{m}}$	—
*did*	*-ed*	*ate*	*-*∅	$\mathrm{T}_{\text{m}}$	none	0
*do*	*-*∅	*ate*	*-*∅	$\mathrm{V}_{\text{m}}^{[\text{O}]}$	none	52
*do*	*-ed*	*ate*	*-*∅	$\mathrm{V}_{\text{m}}^{[\text{O}]}$, $\mathrm{T}_{\text{m}}$	none	0
*did*	*-ed*	*ate*	*-ed*	$\mathrm{T}_{\text{m}}$	$\mathrm{T}_{\text{m}}^{[\text{O}]}$	0
*did*	*-ed*	*eat*	*-ed*	$\mathrm{T}_{\text{m}}$	$\mathrm{V}_{\text{m}}$, $\mathrm{T}_{\text{m}}^{[\text{O}]}$	0
*do*	*-*∅	*ate*	*-ed*	$\mathrm{V}_{\text{m}}^{[\text{O}]}$	$\mathrm{T}_{\text{m}}^{[\text{O}]}$	0
*do*	*-*∅	*eat*	*-ed*	$\mathrm{V}_{\text{m}}^{[\text{O}]}$	$\mathrm{V}_{\text{m}}$, $\mathrm{T}_{\text{m}}^{[\text{O}]}$	0
*do*	*-ed*	*ate*	*-ed*	$\mathrm{V}_{\text{m}}^{[\text{O}]}$, $\mathrm{T}_{\text{m}}$	$\mathrm{T}_{\text{m}}^{[\text{O}]}$	0
*do*	*-ed*	*eat*	*-ed*	$\mathrm{V}_{\text{m}}^{[\text{O}]}$, $\mathrm{T}_{\text{m}}$	$\mathrm{V}_{\text{m}}$, $\mathrm{T}_{\text{m}}^{[\text{O}]}$	0

Indeed, we did find a handful of errors like *did … eated* and one error like *did … ated*. Their extreme rarity, however, is expected given that the likelihoods of each type of mistake, omission of obliteration and flouting of specificity, is already quite low in isolation. The likelihood of a combination of both is therefore even lower and should be close to zero. In addition, as we will further discuss in Sect. 5, the specificity error occurs inconsistently across the different terminals in these errors, a situation that is less likely than a consistent failure across all terminals in a given derivation.

Before we discuss in Sect. 5 the correlation between the frequency of different errors and the type and location of mistake children have to make to produce this error, we briefly show how this system accounts for the errors documented in French causatives.

### Errors in the causative domain

In this section we show how the analysis presented previously extends to the French causative data discussed in Sect. 1. We adopt the Vocabulary entries for the relevant terminals as presented in (1).


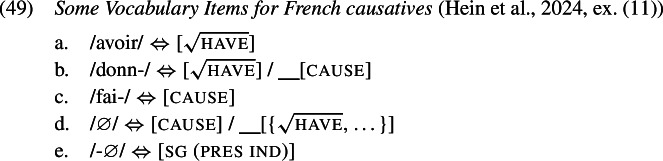
Starting from an underlying structure where the VP is embedded by a Cause head which is in turn embedded by T, we take it that the regular V-to-T movement observed in French (Pollock, 1989) proceeds via Cause if the latter is present. Of course, since we adopt GenHM, this means that there is no actual V-to-Cause-to-T movement. Instead, GenHM applies to Cause and V, and subsequently to the resulting complex and T, eventually producing the complex M-value $\mathrm{V}_{ \text{m}}$–Cause_m_–$\mathrm{T}_{\text{m}}$. This M-value is associated with the syntactic terminals T, Cause, and V, but will only be pronounced in the highest terminal T according to the rules in (19b), and must therefore be delinked from Cause and V at PF, indicated by crossed out association lines in (50).


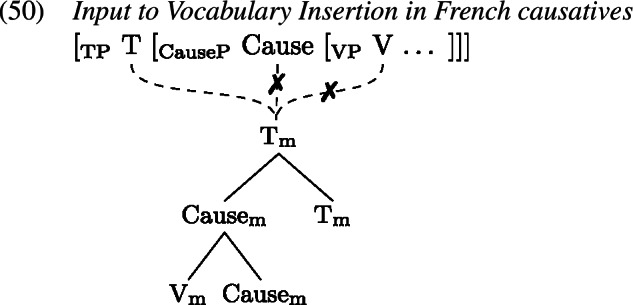
This complex M-value will be the target of Vocabulary Insertion, beginning with the most deeply embedded node, i.e. $\mathrm{V}_{\text{m}}$. In the derivation of a regular lexical causative verb form like *donne* ‘give.3sg’ (51), $\mathrm{V}_{\text{m}}$ will at least contain the root . Thus, both *avoir*^10^ and *donn-* are compatible, but *donn-* is chosen as it is more specific in virtue of its secondary [cause] feature. When insertion targets Cause_m_, both *fai-* and ∅ compete, and ∅ wins because it carries an additional secondary feature in the form of a list of roots, which happens to include . Finally, $\mathrm{T}_{\text{m}}$ is realized by a fitting inflectional exponent, such as the marker for 3rd person singular present tense here.


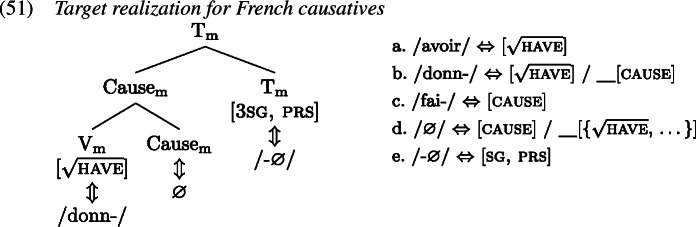
In a later stage of the derivation, we can assume that the zero marker in Cause_m_ is pruned (Embick, 2003, 2010) to enable the host-requiring affix -∅ to attach to *donn-* yielding what is orthographically represented as *donne*.

Let us now turn to the errors, starting with the derivation of the redundant error type in the causative domain (53), based on the example in (52), from Bezinska (2014, 141)’s production study (who analysed these productions as overgeneralisations).


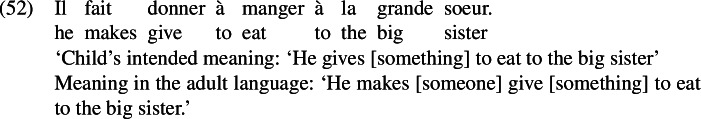
Analogous to the redundant error derivation in the past tense domain, the child manages to select the more specific *donn-* (1b) over the less specific *avoir* (1a) for insertion into $\mathrm{V}_{ \text{m}}$. For insertion targeting Cause_m_, both *fai-* (1c) and ∅ (1d) are viable candidates, since both are compatible with the features in Cause_m_ and its context. Instead of selecting the correct ∅, however, the child inserts *fai-*, thereby disregarding ∅’s secondary features by virtue of which ∅ is more specific than *fai-*. Eventually, $\mathrm{T}_{\text{m}}$ is realized by some inflectional exponent, here 3rd singular present *-*∅ (1e), and the redundant error *fait donner* ‘he gives (lit. he makes give)’ results.^11^


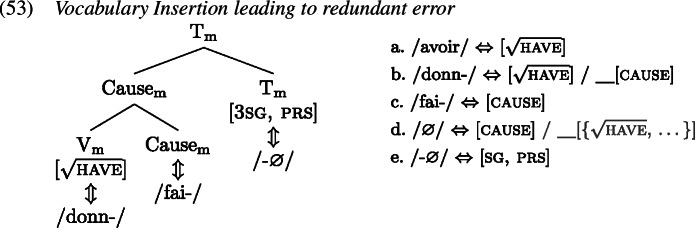
Three things are noteworthy about this derivation. First, in contrast to English past tense redundant errors, the result in the French causatives is a two word expression rather than a single morphologically complex word. We attribute the fact that *fait* constitutes its own word to properties of the Vocabulary entry itself. Second, the bare stem *donn-* is eventually pronounced as an infinitive. We suggest that this is due to a prohibition against bare verb stems in French which leads to the default attachment of the infinitive marker. Third, the finite auxiliary *fai-* precedes the lexical verb *donner* in (-1). We suggest that the unexpected insertion of the free morpheme *fai-* triggers a re-linearization of the complex M-value as part of the general linearization mechanism for the whole syntactic structure.

Just as we saw with the English simple past, we also observe distributive errors in the production of causatives in child French, at least for verbs where the result is lexicalized in a verbal form. So for instance, children sometimes produce *faire avoir* where *donner* is expected, see (54) (see Bowerman, 1982 for similar non-target distributive causative formations in child English).


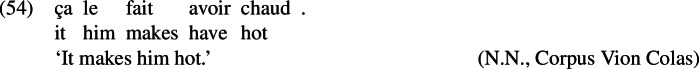
The derivation for the distributive error in (54) is shown in (1). Here, when Vocabulary Insertion targets $\mathrm{V}_{ \text{m}}$, the secondary feature of *donn-* (1b) is neglected leading to the insertion of *avoir* (1a). In the subsequent step at Cause_m_, again the secondary feature of the otherwise more specific item ∅ (1d) is ignored. Instead, the more general item *fai-* (1c) is inserted. Again, $\mathrm{T}_{\text{m}}$ is realized by *-*∅ (1e) as in (0). As with the distributive errors in the English past tense, here, a mistake of secondary feature negligence is made in both positions where it can be made.


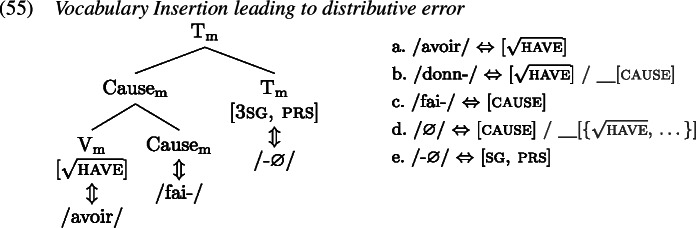
Furthermore, the same way English learners sometimes omit a past exponent, using for instance the present tense form *eat* to express a past eating event, French learners are known to occasionally omit an exponent for cause in the context of a non-causative verb (e.g., *danser* ‘dance’) when they intend to produce a causative statement; see (56) from Sarkar (2002), (cf. also (5b)).


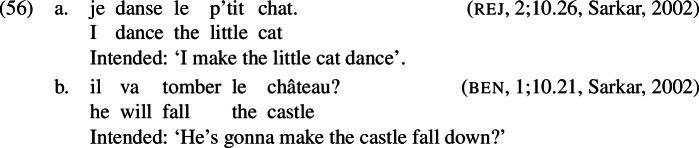
The omission error in, for instance, (56a), can be derived as in (1). Here, the verb *danser* has no lexically causative counterpart. Hence, the item *dans-* (1a) will be inserted into the root  as expected. For the Cause_m_ head, there are two competing items, *fai-* (1b) with no secondary features and ∅ (1c) with a list of roots as a secondary feature, which crucially does not contain . This usually precludes insertion of ∅ in this context. However, when the child ignores this secondary feature, ∅ becomes a candidate for insertion into Cause_m_, which is what happens in (0) leading to an omission error.


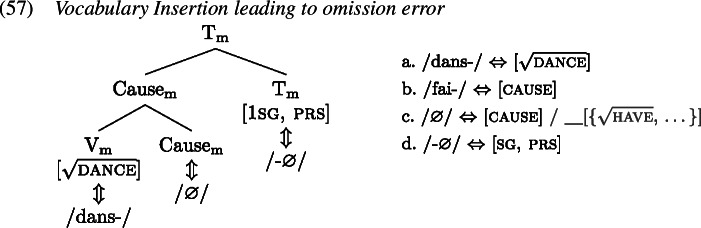
Thus, as in the English past tense in Table 4, the different number and location(s) of secondary feature negligence mistakes in French causatives give rise to four distinct patterns, one target form and three error forms as shown in Table 6.

**Table 6:** Typology of errors in French causatives

$\mathrm{V}_{\text{m}}$	Cause_m_	Error location	Error type
*donn-*	∅	none	target
*dans-*	∅	$\mathrm{V}_{\text{m}}$	omissive
*avoir*	*fai-*	$\mathrm{V}_{\text{m}}$ & $\mathrm{T}_{\text{m}}$	distributive
*donn-*	*fai-*	$\mathrm{T}_{\text{m}}$	redundant

Having illustrated how our analysis of the error patterns in the English past tense can be extended to other domains, we now return to the English past tense domain to discuss the relevance of frequency for error rates.

## Frequency and distribution

### Overall frequencies of error types

As we have argued above, different types of errors occur depending on (i) whether the child disregards secondary feature specificity for insertion (and where), and (ii) whether the child disregards obliteration of an orphaned $\mathrm{T}_{\text{m}}$ node. This, however, says nothing about the vastly different frequencies of occurrence of the different error types. Recall that distributive errors were by far the most frequent error type (1,771 tokens, 1.71%) followed by redundant errors (382, 0.37%). The *did*-type periphrastic error is roughly equally as frequent as the redundant error (365, 0.35%) but much more frequent than the *do*-type (51, 0.05%).

Focusing on the local errors first, i.e. the redundant and distributive ones, we have claimed that they result from the child disregarding secondary features either on $\mathrm{T}_{\text{m}}$ alone or on both $\mathrm{V}_{\text{m}}$ and $\mathrm{T}_{ \text{m}}$, respectively. Prima facie one could expect that the negligence of secondary features for specificity when inserting into a given terminal occurs with a certain (quite low) likelihood *p* between 0 and 1. The likelihood of occurring twice in a row, i.e. for insertion into $\mathrm{V}_{\text{m}}$ and $\mathrm{T}_{\text{m}}$, should therefore be $p^{2}$, which is even lower than *p*. However, distributive errors, where secondary feature negligence occurs twice, are far more frequent than redundant ones, where it only occurs once. We suggest that this is due to a bias for consistency (58) such that a given type of mistake, e.g. secondary feature negligence, is preferably made either on every terminal within a complex M-value or on none. This means that secondary features, if ignored for specificity calculations, strongly tend to be ignored for insertion into both $\mathrm{V}_{\text{m}}$ and $\mathrm{T}_{ \text{m}}$, which results in a distributive error.


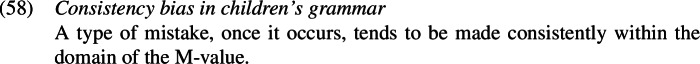
As this bias essentially favours the occurrence of distributive errors, where a one-to-one mapping between form and meaning obtains, it is arguably linked to the bias against one-to-many mappings mentioned in Sects 1 and 4.1 above. While the transparency bias describes children’s more general drive for one-to-one mappings across a range of different domains and phenomena, the Consistency bias in (0) is a more specific instantiation thereof, which covers only those phenomena where a target one-to-many mapping is treated by contextual allomorphy. It thereby essentially constitutes a concrete grammatical implementation of the transparency bias for cases of contextual allomorphy.

Secondary feature negligence may of course also occur on only one terminal, albeit less often. This accounts for the lower rate of redundant errors, which result from secondary feature negligence on $\mathrm{T}_{\text{m}}$. It further predicts that the rate of omission errors should be similar to that of redundant errors, given that they also involve a mistake on only one morphological terminal, namely $\mathrm{V}_{\text{m}}$. Note that (0) makes no claim about the likelihood of a mistake happening, which must be very low. It merely states that in the event of a mistake, the probability of its occurrence on all terminals within the M-value is higher than that of it being restricted to only one terminal.

Turning to the periphrastic errors, where past is marked on the lexical verb either instead of or in addition to being marked on *do*, we have proposed that this is due to the child not obliterating the orphaned $\mathrm{T}_{\text{m}}$ node. This mistake again has a certain (quite low) probability but once it is made, there is a $\mathrm{T}_{\text{m}}$ node present in the lower M-value to be pronounced in V* just as there is one in the higher M-value to be pronounced in T/C. If Vocabulary Insertion proceeds fully regularly, respecting all secondary features, the lower $\mathrm{T}_{\text{m}}$ conditions the insertion of the past tense allomorph *ate* while $\mathrm{T}_{\text{m}}$ itself is realized by ∅ (cf. example (48) above). In the higher M-value, *did* is selected for $\mathrm{V}_{ \text{m}}$ while $\mathrm{T}_{\text{m}}$ is realized by ∅. Thus, when obliteration is omitted but no further (secondary feature negligence) mistakes are made, we get an output where past tense is marked on the lexical verb as a portmanteau and on the light verb as *did*. A derivation without any secondary feature mistakes is the norm in non-periphrastic constructions as evidenced by the 97.19% target rate. This leads us to expect that the majority of derivations with an obliteration mistake do not also involve a secondary feature mistake. Consequently, the majority of periphrastic errors should be of the type *did ate*. Indeed, this is by far the most frequent periphrastic error in our sample (356 tokens, 0.34% of all tokens, 85.37% of periphrastic errors).

All other periphrastic errors given in Table 5 involve an obliteration mistake plus at least one additional secondary feature mistake, i.e. negligence of secondary features on the lower M-value (giving *did eated, did ated*), the higher M-value (giving e.g. *do ate, doed ate*), or both (giving e.g. *do eated, doed ated*, etc). As the probability of an obliteration mistake and that of secondary feature negligence are already quite low in isolation, the combined probability of both errors is even smaller. Therefore, periphrastic errors other than *did ate* are expected to be infrequent. Note that the omission of obliteration and secondary feature negligence constitute two distinct types of mistakes. The Consistency bias, as formulated in (58), therefore does not favour their cooccurrence as it holds only for mistakes of the same type. Nonetheless, in cases where the mistakes do occur in combination, we would expect the preference for distributive over redundant errors (cf. Table 3) within each M-value to prevail. This is because (58) favours wholesale negligence of secondary features inside a given M-value (but not necessarily in both M-values) once a mistake of secondary feature negligence occurs in that M-value. Indeed, this seems to be the case, as suggested by the fact that there is only one error of the *did ate-d* type, where obliteration omission is accompanied by a redundant error on the lower M-value, whereas there are eight errors of the *did eat-ed* type, where obliteration omission is followed by a distributive error on the lower M-value.^12^

### Error rates by lexical verb

**Fig. 2:**
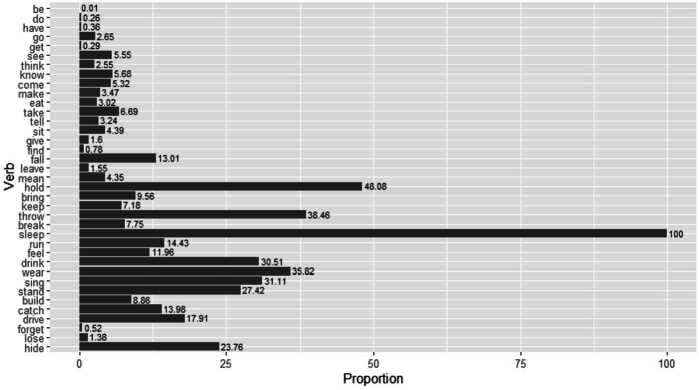
Proportion of errors (redundant, distributive) by verb ordered by input frequency from most frequent verb (be) to least frequent (hide).

The frequency of errors need not necessarily be the same for each lexical verb. It has been observed that irregular verbs that frequently occur in the child’s input tend to be less prone to errors than those occurring less frequently (Maslen et al., 2004, Räsänen et al., 2014) with phonological factors blurring the correlation to some degree, as, for example, past forms marked only by a stem vowel change (*hold*∼*held*) are more error-prone than those that have an additional consonantal past marking (*keep*∼*kep-t*). This relation between input frequency and error rate is also found in our data albeit in a somewhat blurred form. Figure 2 shows the proportion of errors for each verb, focussing on redundant and distributive ones only, with the verbs ordered by input frequency.^13^ In this graph, the verb *hide*, for instance, which is the least frequent one in the documented input of the children in CHILDES, is produced as a distributive (*hide-d*) or redundant (*hidd-ed*) error in 23.76% of the children’s total productions of a past tense form of *hide*.

**Fig. 3:**
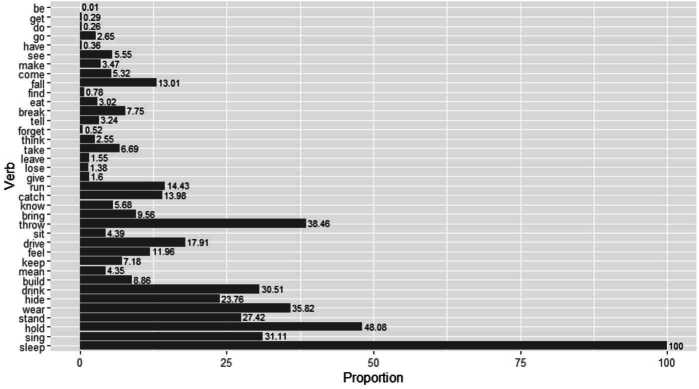
Proportion of errors (redundant, distributive) by verb ordered by output frequency from most frequent (be) to least frequent (sleep).

The frequency ranking in this plot was obtained by Sketch Engine’s wordlist function as described in Sect. 2.1 filtering English CHILDES for all adult participant roles. We take this as a proxy for the actual frequencies of different lexical verbs in child-directed speech. With the exception of a few outliers we can see that the higher-frequency verbs in the top half of the plot have a relatively low error proportion below 10%. Many of the lower-frequency verbs in the bottom half, by contrast, show error rates far above 10%. To confirm this impression, a logistic mixed model was fit to the data, with standardized log input frequency as a fixed effect and child and verb as random effects. Distributive and redundant errors were coded as 1 and targets as well as periphrastic errors as 0. A statistically significant negative effect of input frequency on error production was found, meaning that higher frequent verbs are less prone to errors than lower frequent ones (*β̂* = −1.6, SE = 0.24, *z* = −6.52, *p*<.001). Interestingly, the correlation becomes a little stronger if, instead of input frequency, we order the verbs by their number of occurrence in our data, i.e. by output frequency. This plot is provided in Fig. 3, where the verb *sleep*, for example, is least frequently produced in a past tense context by the children in CHILDES and 100% of these productions are distributive (*sleep-ed*) or redundant (*slept-ed*) errors. Again, we fit a logistic mixed model to the data in Fig. 3, with standardized log frequency as a fixed effect and by-child and by-verb varying intercepts included. Distributive and redundant errors were coded as 1 and targets as well as periphrastic errors as 0. We found that frequency has a statistically significant negative effect on error production, that is, higher frequent verbs are less likely to be produced with an error (*β̂* = −1.3, SE = 0.12, *z* = −11.15, *p*<.001).

A reason for the slightly stronger effect with output frequency might be that input frequency subsumes past, participial, and crucially also present tense tokens, which are not informative with regard to the past tense formation of a verb. Our data in Fig. 3 only contains past tense forms (albeit partly erroneous ones) and therefore provides a better proxy for the actual frequency of each verb in past tense in regular speech.

We suggest that this correlation between frequency and error rate might be due to the fact that the probability of secondary feature negligence for a given Vocabulary Item correlates inversely with the stability of the representation of the secondary feature within the lexical entry of the Vocabulary Item.^14^ That is, the more consolidated the secondary feature is, the less likely it is to be disregarded upon Vocabulary Insertion. The representational stability of a secondary feature is in turn dependent on the frequency with which a Vocabulary Item is encountered in the relevant environment by the child; in other words, it is dependent on the frequency of the relevant form in the child’s input. Thus, the more frequent an irregular verb in past tense environment is in child-directed speech, the more stable the representation of its past tense form will be, including secondary features, and the less likely it is to be subject to secondary feature negligence. The same holds for the secondary feature list of roots that is part of the zero past marker’s Vocabulary entry. The more it cooccurs with a specific root in the child’s input, the more stable is this root’s presence on the list of roots.

**Fig. 4:**
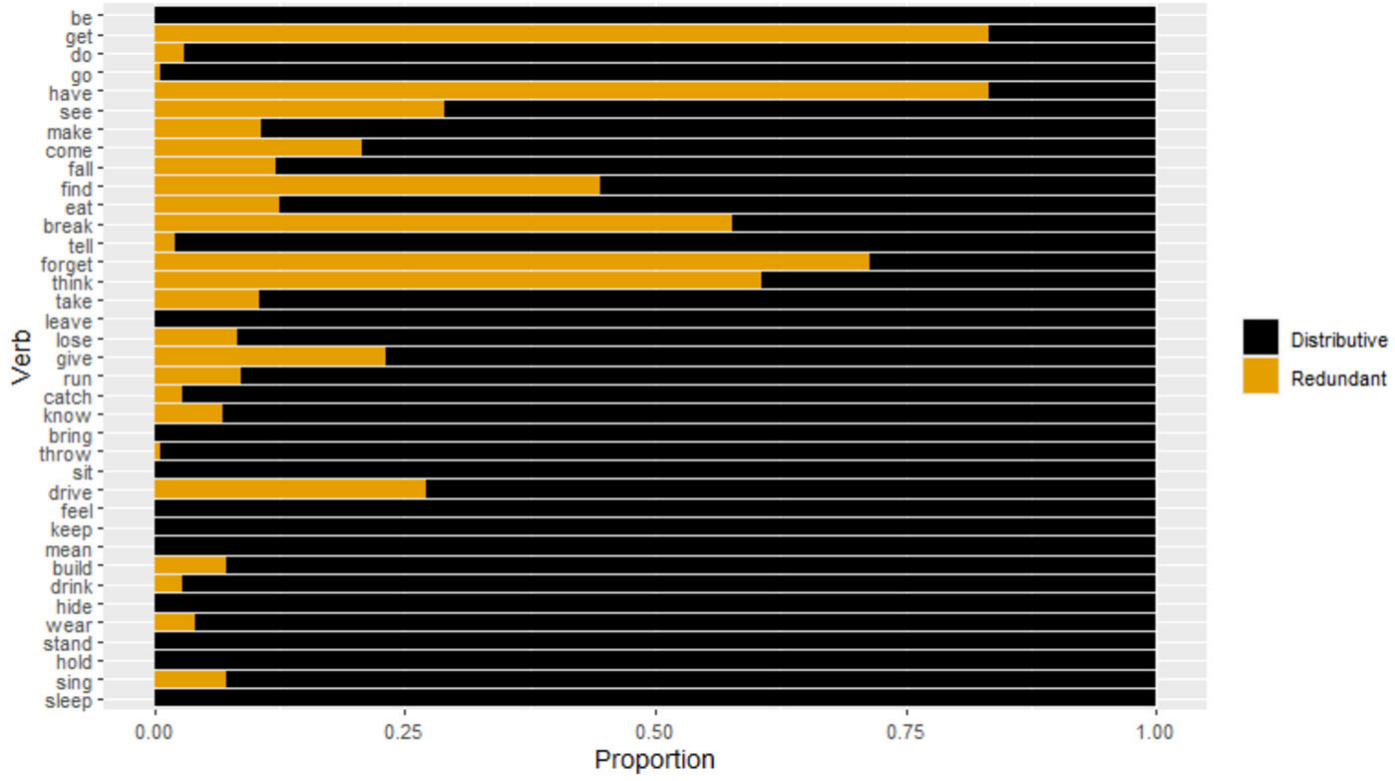
Proportion of distributive vs. redundant errors within all local errors by verb ordered by output frequency (colour figure online)

As for the proportions of different error types within all local errors for each verb, we find that they vary. While for most verbs the proportion of distributive errors is higher than that of redundant errors, a few verbs, i.e. *get*, *have*, *break*, *forget*, and *think*, show a majority of redundant errors (Fig. 4). In general, the proportion of redundant errors seems to be higher for more frequent verbs than for less frequent verbs. This impression is confirmed by fitting a logistic mixed effects model to the data, with standardized log output frequency as the fixed effect and child and verb as random effects. Targets and periphrastic errors were excluded from the data. Redundant errors were coded as 1 and distributive ones as 0. There is a statistically significant positive effect of frequency on redundant error, meaning that higher-frequency verbs appear with a higher proportion of redundant errors than lower-frequency ones (*β̂* = 1.0, SE = 0.28, *z* = 3.67, *p*<.001).

The fact that most verbs still show more distributive errors than redundant ones is in line with the Consistency bias. Concerning the verbs that exhibit more redundant errors, there are three ways one could think of this. First, the relatively high rate of redundant errors could simply be an artifact of the data. Second, there might be an additional (possibly phonological) factor at play that skews the distribution towards redundant errors for the verbs in question. Third, it might be possible that the Consistency bias is not equally strong across all verbs but is relativized to each lexical verb such that it is weaker the more frequent a verb is.

## Conclusion

We have provided a comprehensive contemporary corpus study of overregularization and overtensing errors in the acquisition of English. We distinguished three main types of errors, distributive, redundant and periphrastic ones, where the latter subclassify into *did*-type and *do*-type errors. While error rates for all errors are quite low, which is in line with most previous work (Kuczaj, 1977, 1978; Marcus et al., 1992), they peak at the same age of around 30 months and subside by the age of 100 months. Distributive errors constitute by far the most frequent type while redundant and periphrastic errors are less frequent. Again, this aligns with previous findings (Kuczaj, 1977, 1978; Marcus et al., 1992). We proposed a unified analysis of the errors within the framework of Distributed Morphology combined with Generalized Head Movement which derives all errors from two underlying mistakes: negligence of secondary features and omission of obliteration, and their interaction. The first type of mistake in particular can be understood as a means for the child to achieve a one-to-one mapping of the form of an exponent and its meaning through the reduction of the number of features associated with a given Vocabulary Item. It thereby constitutes an implementation of the more general bias for transparent form-meaning relations observed in acquisition (Slobin, 1985; Brighton et al., 2005; van Hout, 2008; Guasti et al., 2023). Further, we presented some ideas about how to account for the different error rates of the three error types as well as how these differ depending on the verb’s frequency.

## Footnotes

1 Corpora were not searched for *buy* as for some reason this verb was erroneously not included in the search query’s list of verbs. This was only noticed while writing this paper.

2 Note that the data available through CHILDES is not evenly distributed across all ages, with most of it coming from 2- to 3-year-old children. Absolute error numbers will therefore always be larger at this stage than at later ones. Figure 1 reports relative error numbers in the form of error rates and thus circumvents this potential confound.

3 We chose to stay faithful to the original corpus data, including comments/corrections by the annotators, which occur mostly in square brackets. Occasionally, errors were only documented in the annotator’s comments, while other times the correct target form was given in the annotator’s comment. The relevant verb form is always boldfaced throughout this paper.

4 If we take the number of occurrences of the lemma *do* as a basis (13,069), the error rate for periphrastic errors increases by about a factor 10 to 3.2%. This is likely still an underestimation as this basis also contains cases where *do* occurs as a main verb.

5 Arnon (2009) found a similar distribution in errors with irregular plurals in English, e.g. *foots*∼*feets*, where the distributive errors (*foots*) are three times more frequent than the redundant ones (*feets*).

6 As the order in which the features appear in the complex is not fixed by GenHM, we will assume here that the lexical items themselves, e.g. *-ed*, are specified for whether they are prefixes or suffixes and thus determine the order upon insertion. For the English past tense, this results in structures well-known from classical head movement accounts, where structurally lower heads are left-adjoined to structurally higher ones. In other cases, additional factors may influence linear order (cf. the discussion of French causatives in Sect. 4.3).

7 Auxiliaries are always pronounced in T since they are weak in both languages. The strength of V has no effect on pronunciation anymore, as V constitutes its own trivial head chain given that auxiliaries never bear a [hm] feature and themselves satisfy the [hm] feature on T (Arregi & Pietraszko, 2021, 247).

8 A reviewer notes that the negligence of the secondary feature of /ate/ leads to a situation where the root  freely maps to both /eat/ and /ate/ in a one-to-many fashion. This seems to be in conflict with the children’s drive towards one-to-one mappings. However, in order to evaluate global mappings such as the one pointed out by the reviewer, children would have to check their entire mental lexicon for forms that potentially map to the same particular root. We suggest that children only master this at more advanced stages of their cognitive development. Therefore, they first implement the one-to-one bias at the local level of the individual lexical items.

9 Since our original search was run for past tense forms, any tokens we caught of regular verbs with a past tense meaning without the overt /-ed/ marker were only incidental. In addition, these mistakes must have been noticed and also marked by the transcriber of the original spoken data (typically by adding “[* 0ed]” after the relevant verb), which is not a given. We extracted these errors from our data sample by searching for “[* 0ed]”. The fact that these data all stem from only two corpora is probably because only in these corpora those errors were noticed and the marking convention “[* 0ed]” was adopted.

10 We use the form *avoir* here as a stand-in for the actual stem of the respective lexeme meaning ‘have’ for it is notoriously suppletive.

11 Note that the phonologically zero ending in the singular for *fai-* is orthographically marked by *-s* in 1st and 2nd person and as *-t* in 3rd person.

12 The relatively high number of *do ate* type tokens within the periphrastic errors (51, 12.26% of periphrastic errors), where past is left unmarked in the higher M-value, is somewhat surprising under this perspective. We currently do not have an explanation for this.

13 We exclude periphrastic errors from this discussion because they do not involve the negligence of a secondary feature that is directly tied to the verb lemma but instead are generated by a more general failure to obliterate a morphological terminal. As neither the terminal nor obliteration is linked to the identity of the verb lemma, an interaction with frequency is not expected. That said, including periphrastic errors does not change the statistical significance of the correlations observed in the ensuing discussion.

14 This stability could be understood as the secondary feature having a gradient representation, a concept that is usually employed for phonological representations (Smolensky & Goldrick, 2016; Zimmermann, 2018, 2019, 2021), but has also been applied to (morpho-)syntactic representations under the notion of grammatical strength (Müller, 2019; Müller et al., 2022; see Hsu, 2022 for an overview).
